# Supplementary material for: Systematic review of Plasmodium falciparum and Plasmodium vivax polyclonal infections: Impact of prevalence, study population characteristics, and laboratory procedures
Source: PLoS One. 2021 Jun 11;16(6):e0249382. doi: 10.1371/journal.pone.0249382 (PMC8195386; doi:10.1371/journal.pone.0249382)
Supplement: S1 Fig — (DOC) [file pone.0249382.s002.doc]

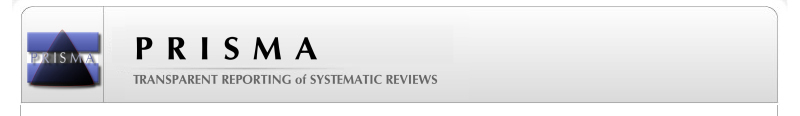
**PRISMA 2009 Flow Diagram**

**Screening**

**Included**

**Eligibility**

**Identification**

Records identified through database searching
(n = 695 )

Additional records identified through other sources
(n = 0 )

Records after duplicates removed
(n = 659 )

Records screened
(n = 659 )

Records excluded
(n = NA )

Full-text articles assessed for eligibility
(n = 259 )

Full-text articles excluded, with reasons
(n = 106 )

Studies included in qualitative synthesis
(n = 153 )

Studies included in quantitative synthesis (meta-analysis)
(n = NA )
